# Enhancement of bleomycin production in *Streptomyces verticillus* through global metabolic regulation of *N*-acetylglucosamine and assisted metabolic profiling analysis

**DOI:** 10.1186/s12934-020-01301-8

**Published:** 2020-02-13

**Authors:** Hong Chen, Jiaqi Cui, Pan Wang, Xin Wang, Jianping Wen

**Affiliations:** 1 Key Laboratory of Systems Bioengineering (Ministry of Education), Tianjin University, Tianjin, 300072 People’s Republic of China; 2 SynBio Research Platform, Collaborative Innovation Center of Chemical Science and Engineering (Tianjin), School of Chemical Engineering and Technology, Tianjin University, Tianjin, 300072 People’s Republic of China

**Keywords:** Bleomycin, GlcNAc, DasR, GDP-mannose, Metabolic profiling

## Abstract

**Background:**

Bleomycin is a broad-spectrum glycopeptide antitumor antibiotic produced by *Streptomyces verticillus*. Clinically, the mixture of bleomycin A2 and bleomycin B2 is widely used in combination with other drugs for the treatment of various cancers. As a secondary metabolite, the biosynthesis of bleomycin is precisely controlled by the complex extra-/intracellular regulation mechanisms, it is imperative to investigate the global metabolic and regulatory system involved in bleomycin biosynthesis for increasing bleomycin production.

**Results:**

*N*-acetylglucosamine (GlcNAc), the vital signaling molecule controlling the onset of development and antibiotic synthesis in *Streptomyces*, was found to increase the yields of bleomycins significantly in chemically defined medium. To mine the gene information relevant to GlcNAc metabolism, the DNA sequences of *dasR*-*dasA*-*dasBCD*-*nagB* and *nagKA* in *S. verticillus* were determined by chromosome walking. From the results of Real time fluorescence quantitative PCR (RT-qPCR) and electrophoretic mobility shift assays (EMSAs), the repression of the expression of *nagB* and *nagKA* by the global regulator DasR was released under induction with GlcNAc. The relief of *blmT* expression repression by BlmR was the main reason for increased bleomycin production. DasR, however, could not directly affect the expression of the pathway-specific repressor BlmR in the bleomycins gene cluster. With at the beginning of bleomycin synthesis, the supply of the specific precursor GDP-mannose played the key role in bleomycin production. Genetic engineering of the GDP-mannose synthesis pathway indicated that phosphomannose isomerase (ManA) and phosphomannomutase (ManB) were key enzymes for bleomycins synthesis. Here, the *blmT*, *manA* and *manB* co-expression strain OBlmT/ManAB was constructed. Based on GlcNAc regulation and assisted metabolic profiling analysis, the yields of bleomycin A2 and B2 were ultimately increased to 61.79 and 36.9 mg/L, respectively.

**Conclusions:**

Under GlcNAc induction, the elevated production of bleomycins was mainly associated with the alleviation of the inhibition of BlmT, so *blmT* and specific precursor synthesis pathways were genetically engineered for bleomycins production improvement. Combination with subsequent metabolomics analysis not only effectively increased the bleomycin yield, but also extended the utilization of chitin-derived substrates in microbial-based antibiotic production.

## Introduction

Bleomycin is a member of glycopeptide antibiotics that was first isolated from the fermentation broth of *S. verticillus* by Umezawa et al. [1]. The bleomycin gene cluster and its structure clearly indicate that a hybrid peptide-polyketide skeleton and a disaccharide unit together constitute bleomycins with different C-terminal amines [2]. A mixture of bleomycin A2 and B2, marketed as Blenoxane^®^, is administered by injection in clinic to treat tumors, notably germ cell tumors, head and neck cancer and cervical carcinoma [3]. Although fermentation medium optimization and manipulation of the pathway-specific repressor *blmR* have been suggested to be useful for enhancing bleomycin production [4–6], the lower yields and complex nutrient demands in rich medium have been major difficulties in the industrial production of bleomycins.

*Streptomyces* species inhabit specific environments with changing nutrient availability, and their morphological development is tuned to coincide with the synthesis of secondary metabolites, including antibiotics. Under starvation, multiple regulatory networks are employed by *Streptomyces* to make a decision whether to continue vegetative growth or form spores. Once it enters the spores development, a series of genes are transcribed, which activate the synthesis of antibiotics and morphological differentiation [7, 8]. The onset of antibiotic synthesis not only depends on the expression of the specific biosynthetic gene clusters, but is also affected by the regulation of intracellular response mechanism of environmental status [9]. GlcNAc, the main component of chitin, is considered to be a signaling molecule with different effects on spore development and antibiotic yields in *Streptomyces* under nutrient-rich/poor conditions [10, 11]. Actually, the regulation of the GntR/HutC family repressor DasR has been found to be essential for the development and morphogenesis process, which controls the expression of genes involved in chitin/GlcNAc metabolism, the development-related phosphoglucosyltransferase system (PTS), stress response and nearly all the known antibiotic biosynthesis pathways [10, 12–15]. In vivo, DasR binds to a conserved element in the promoter region of its target genes and represses their expression. However, the metabolites *N*-acetylglucosamine 6-phosphate (GlcNAc-6P) and glucosamine-6-phosphate (GlcN-6P) in GlcNAc catabolism would competitively bind to DasR, which is then released it from the target sites [14–16]. It has been reported that the addition of GlcNAc to a niche simulates a signal similar to cell wall lysis under nutritional deficiency, which is delivered and sensed by *Streptomyces* to initiate sporulation and secondary metabolism [17]. Therefore, not only does GlcNAc or chitin provide a source of carbon and nitrogen for strain growth, but the GlcNAc-induced regulation of antibiotic synthesis should be utilized to improve the bleomycin production in *S. verticillus*.

After the onset of antibiotic synthesis, the supply of limiting precursors is another key factor for improving antibiotic yield [18]. Mannosylated glycans are common precursors of various glycoproteins and antibiotics including cell envelope polymers, polyenes, bleomycins and mannopeptimycins. Mannose can be sequentially converted by phosphomannose isomerase (ManA), phosphomannomutase (ManB), and GDP-mannose pyrophosphorylase to form GDP-mannose. During bleomycin biosynthesis, GDP-mannose and its isomer GDP-gulose are attached to the bleomycin aglycone through glycosylation, which is responsible for the cellular recognition, uptake and metal ions chelation of bleomycins [19]. As the final step in the biosynthesis of most glycopeptide antibiotics, the genetic engineering of glycosylation could not only result in hyper-susceptibility to antibiotics and diverse derivatives of natural products, but its reaction efficiency usually dictates the ultimate yield of an antibiotic [20–23]. In addition to the glycosyl moieties, the hybrid peptide-polyketide skeleton of bleomycin is directly synthesized by nine common amino acids [24], which indicate that the intracellular primary metabolic pathways of *S. verticillus* might also play a dominant role in bleomycin biosynthesis. To further increase the yield of bleomycin, it is necessary to fully understand the changes of global intracellular metabolites and identify key modules that are associated with the synthesis of bleomycins. Metabolic profiling analysis is widely employed for the identification of biomarkers and modules associated with target product synthesis. This approach has been used to increase the outputs of natural products and bioenergy [25, 26].

In this study, the signaling molecule GlcNAc greatly initiated the bleomycin biosynthesis in liquid medium. To elucidate the GlcNAc induction mechanism, the effects of the global transcription factor DasR on GlcNAc metabolism and bleomycin synthesis were investigated by RT-qPCR and EMSAs. The elevated expression of the *blmT* gene under GlcNAc induction was found to be the main cause of the improvement of bleomycin yield. To further increase bleomycin production under GlcNAc induction, gene *blmT* was overexpressed and the supply of the precursor GDP-mannose was also enhanced by genetically engineering the mannose catabolism pathway. Assisted by comparative metabolic profiling analysis, the production of bleomycin A2 and B2 was respectively improved to 61.79 and 36.9 mg/L in the GlcNAc-derived chemically defined medium.

## Materials and methods

### Strains, plasmids and bleomycins fermentation

*Streptomyces verticillus* ATCC 15003 purchased from American type culture collection (ATCC) was used as the bleomycins-producing wild-type strain. All the strains and plasmids used in this study were listed in Additional file 1: Table S1. The spores of *S. verticillus* were preserved on ISP4 solid medium [27] with the addition of 0.5 g/L yeast extract and 1 g/L tryptone and minimal medium (MM) solid medium, respectively. MM solid medium contained 0.5 g/L l-asparagine, 0.5 g/L K_2_HPO_4_, 0.2 g/L MgSO_4_·7H_2_O, 0.01 g/L FeSO_4_·7H_2_O and 6 g/L mannitol. For bleomycin production, the mature spores were cultured for 48 h in seed medium containing 20 g/L soybean powder, 25 g/L soluble starch, 5 g/L glucose, 1 g/L K_2_HPO_4_, 0.5 g/L ZnSO_4_, 0.1 g/L CuSO_4_·5H_2_O and 2 g/L CaCO_3_. Aliquots comprising 4 mL of the pre-culture were transferred and cultivated for 7 days in complex fermentation medium, modified ISP4 liquid medium, medium I and medium II, respectively. The complex fermentation medium was the same as described in the previous literature [4]. The modified ISP4 liquid medium contained 20 g/L soluble starch, 20 g/L dextrin, 1 g/L KH_2_PO_4_, 1 g/L MgSO_4_·7H_2_O, 1 g/L NaCl, 3 g/L KNO_3_, 0.001 g/L FeSO_4_·7H_2_O, 0.001 g/L MnCl_2_, 0.001 g/L ZnSO_4_·7H_2_O and 5 g/L 3-Morpholinopropanesulfonic acid (MOPS). Medium I was formed by adding 5 g/L of GlcNAc to modified ISP4 liquid medium. Medium II was composed of 10 g/L mannose, 5 g/L chitosan, 20 g/L GlcNAc, 40 g/L dextrin, 1 g/L KH_2_PO_4_, 1 g/L MgSO_4_·7H_2_O, 1 g/L NaCl, 6 g/L KNO_3_, 0.001 g/L FeSO_4_·7H_2_O, 0.001 g/L MnCl_2_, 0.001 g/L ZnSO_4_·7H_2_O and 5 g/L MOPS. The pH of the seed medium and fermentation medium was adjusted to 6.5 with 3 mol/L NaOH solution. *Escherichia coli* DH5α and S17-1 were cultured in Luria–Bertani medium (LB).

Due to the water-solubility characteristic of bleomycins, the fermentation broth of *S. verticillus* was centrifugated at 12000 r/min for 10 min, following by filtration through a 0.22 μm micropore membrane. The supernatant was subjected to High Performance Liquid Chromatography (HPLC) 1200 equipped with a Venusil MP-C18 column (5 μm, 250 mm × 4.6 mm). The HPLC program was described in previous literature [4].

### Gene identification involved in GlcNAc metabolism

To extract genomic DNA used for chromosome walking, the mature spores were suspended in Tryptic Soy Broth (TSB) medium (BD Biosciences, America) with the addition of 0.4% glycine and 10% sucrose (m/v). After 48 h of cultivation, 1 mL of culture was taken to collect the mycelium by centrifugation, which was then used to extract genomic DNA according to the protocol of the TIANamp Bacteria DNA Kit (Tiangen, Beijing). To clone the conserved DNA fragments of genes related to GlcNAc metabolism, a potential bleomycins producer *Streptomyces mobaraensis* DSM 40847 [28], which previously was classified as *Streptoverticillium* [29] and used as reference strain. The amino acids and DNA sequences of gene *dasR* (Gene ID: WP004937563.1), *manA* (Gene ID: WP004953455.1) and *nagA* (Gene ID: WP004954541.1) in *S. mobaraensis* DSM 40847 were downloaded from the National Center for Biotechnology Information (NCBI) and submitted to protein/nucleotide BLAST to find the corresponding target sequences with high homology in *Streptomyces*. Then, multiple sequence alignments were performed using Clustal Omega (https://www.ebi.ac.uk/Tools/msa/clustalo/) to identify highly conserved amino acids/nucleotides regions, which were used for designing degenerate primers. The PCR using genomic DNA of *S. verticillus* ATCC 15003 as template with degenerate primers were performed to amplify the conserved DNA fragments of *dasR*, *manA* and *nagA*. Based on the conserved DNA regions, the SiteFinding PCR was performed according to the published protocol with minor modifications [30, 31]. A detailed schematic diagram was presented in Additional file 1: Fig. S1. A 20 μL SiteFinding PCR mixture contained 10 μL 2× Phanta Max Buffer, 0.5 μL dNTP Mix (10 mM each), 1 μL SiteFinder (10 μM), 0.5 U Phanta Max Super-Fidelity DNA Polymerase (Vazyme Biotech Co., Ltd, Nanjing, China) and 50–100 ng template DNA. The *Not*I site and six degenerate nucleotides (NNNNNN) of SiteFinder-1 from Tan and colleagues’ protocol [31] were replaced by *Xba*I and NNNCGG, and the final SiteFinder (5′ CACGACACGCTACTCAACACACCACCTCGCACAGCGTCCTCAAtctagaNNNCGGGCGC 3′) in our study was used for the efficient amplification of high GC template DNA. After the SiteFinding PCR, the first nested PCR was employed to amplify the target DNA fragment. A 5 μL mixture containing 2 μL of 10 μM SFP1, 1 μL of 10 μM gene specific primer 1 (GSP1) and 2 μL of 2× Phanta Max Buffer was added to the 20 μL SiteFinding PCR reaction mixture. The largest DNA fragment among the amplification products of the first nested PCR was used as the template, the second round of nested PCR was performed and the mixture (50 μL) contained 25 μL of 2× Phanta Max Buffer, 1 μL of dNTP Mix (10 mM each), 2 μL of SFP2 (10 μM), 2 μL of GSP2 (10 μM), 1 U of Phanta Max Super-Fidelity DNA Polymerase and an appropriate volume of ddH_2_O. The primers used for the SiteFinding and nested PCR were presented in Additional file 1: Table S2. To construct plasmids for sequencing, the amplification products from the second round of nested PCR were purified using FastPure Gel DNA Extraction Mini Kit (Vazyme Biotech Co., Ltd, Nanjing, China). After double digestion with the restriction enzymes PstI and XbaI, the fragments were cloned into the plasmid pUC18 and then transformed into *E. coli* DH5α. The corresponding plasmids were sequenced by Genewiz (Suzhou, China) and the sequences were deposited into the GenBank. The GenBank accession numbers of *dasR*-*dasA*, *dasB*-*dasC*-*dasD*-*nagB*, *manA*, *manB* and *nagK*-*nagA* were MN380024, MN380025, MN380026, MN380027 and MN380028, respectively. The DNA sequences were translated to corresponding amino acids sequences using the Bacterial, Archaeal and Plant Plastid Code (transl_table = 11) on NCBI (https://www.ncbi.nlm.nih.gov/orffinder/).

### Construction of gene engineering strains

For the overexpression of single gene, the whole nucleotide sequences of gene *manA* (GenBank: MN380026), *manB* (GenBank: MN380027), *blmC* (GenBank: AAG02371.1), *blmD* (GenBank: AAG02370.1), *blmE* (GenBank: AAG02369.1), *blmF* (GenBank: AAG02362.1), *blmG* (GenBank: AAG02361.1) and *blmT* (GenBank: AAG02352.1) were amplified by PCR using corresponding primer pairs (Additional file 1: Table S2), and were respectively ligated into the integrative plasmid pIB139 using the ClonExpress II One Step Cloning Kit (Vazyme, Nanjing, China). The recombinant plasmids were introduced into *E. coli* S17-1 for conjugal transfer into *S. verticillus* ATCC 15003 according to the literature [32]. After screening for positive transformants, genomic DNA extraction and sequencing, the overexpression strains of *manA*, *manB*, *blmC*, *blmD*, *blmE*, *blmF*, *blmG* and *blmT* were constructed and designated as OManA, OManB, OBlmC, OBlmD, OBlmE, OBlmF, OBlmG and OBlmT, respectively.

For the construction of bleomycin overproduction strain, the encoding sequences of *blmT*, *manA* and *manB* were amplified using the corresponding primer pairs listed in Additional file 1: Table S2. The PCR product of *manA* was cut with NdeI and PstI, which was then cloned into pUC18 to generate pUC18/manA. After the digestion with PstI and XbaI, the segment of *manB* was ligated with pUC18/manA to obtain the plasmid pUC18/manA/manB. To construct the *manAB* overexpression plasmid, the fusion fragment of *manA* and *manB* was digested by NdeI and XbaI, which was then cloned into the pIB139 using T4 DNA ligase (Thermo Fisher Scientific, USA) to generate pIB139/manA/manB. The PCR product of gene *blmT* was digested by XbaI and NotI, which was cloned into pIB139/manA/manB to generate pIB139/BlmT/ManAB. After the verification by restriction enzyme digestion and sequencing, the correct recombinant plasmid was introduced into *S. verticillus* ATCC 15003 by conjugal transfer mediated by *E. coli* S17-1. The overproduction strain OBlmT/ManAB was selected via apramycin resistance screening and sequencing. Besides, strains DBlmR (BlmR deletion strain) and DBlmT-ΔBlmR (BlmR deletion and BlmT overexpression strain) were constructed based on the protocol described in our previous literature [4].

### RT-qPCR

To study the effect of GlcNAc induction on the expression of *das* genes and bleomycins synthesis genes, 4 mL of pre-culture of *S. verticillus* ATCC 15003 was inoculated into the modified ISP4 fermentation medium. After the cultivation for 36 h, GlcNAc with a final concentration of 5 g/L was added into the fermentation medium. RNA samples were collected by centrifugation (4 °C and 8000×*g*) at 0 h and 2 h after the GlcNAc addition. RNA extraction and cDNA synthesis were performed as described in previous literature [4]. The RT-qPCR was carried out using LightCycler^®^ 480 SYBR Green I Master (Roche, Switzerland) in a LightCycler 480 (Roche, Switzerland). The primer pairs used in RT-qPCR were listed in Additional file 1: Table S2. The 20 μL qRT-PCR reaction mixtures contained 2 μL of cDNA, 1 μL of forward/reverse primers, 10 μL of 2× Master Mix and 6 μL of PCR grade water. The same volume (2 μL) of reverse transcription mixture containing a DNase I-treated RNA sample without reverse transcriptase was used as negative control to exclude interference from residual genomic DNA. The 16S RNA was used as internal control, and the expression levels of target genes in medium I were evaluated by normalizing them to those of *S. verticillus* cultured in modified ISP4. Three independent biological replicates were conducted in our study.

### Protein purification and EMSAs

For the production of 6 × His-tagged DasR, the DNA sequence of DasR without stop codon was amplified using primers PdasR/F and PdasR/R, in which the 6 × His tag was introduced by PdasR/R. After the digestion by NdeI and BamHI, the fragment of *dasR* was cloned into plasmid pET-3c. The obtained pET-3c/DasR was transformed into host *E. coli* BL21 Rosetta (DE3) for DasR expression. The *E. coli* BL21 Rosetta (DE3)/pET-3c/DasR were cultivated at 37 °C for 2–3 h. After the optical density at 600 nm reached 0.6–0.7, a final concentration of 0.2 mM iso-propyl-β-d-thiogalactoside (IPTG) was added to induce the production of DasR. The purification of DasR was performed as described as previous literature [4] and SDS-PAGE profile was showed in Additional file 1: Fig. S2. Besides, the 6 × His-tagged BlmR was also purified [4] and used to verify the effect of GlcN-6P on the binding of BlmR to its target site. The partial DNA sequences spanning the potential *dre* sites in the promoters of *dasA*, *nagB* and *nagKA* were acted as probes in EMSAs (Additional file 1: Fig. S3), in which all the single-strand DNA and its complementary fragments were chemically synthesized and labeled with Cy5 at 5′-end. After mixing equally in the annealing buffer, the double-stranded probes P_DasA_, P_NagB_ and P_NagKA_ were respectively obtained by touchdown PCR. Furthermore, the whole promoter probe P_BlmR_ and BlmR binding sequence probe P_BlmR_-1 were constructed according to Chen et al. [4], both were present in Additional file 1: Fig. S3. DasR and various probes were incubated for 30 min in EMSAs buffer, the mixture was then measured by 6% native polyacrylamide gels. To investigate whether GlcNAc metabolites could induce the dissociation of DasR-DNA and BlmR-DNA complexs, the different concentrations of GlcNAc and GlcN-6P were used as additives in the EMSAs system. Besides, the reaction mixture without inducers was used as control group.

### Gas chromatograph mass spectroscopy (GC–MS) determination and statistical analysis

To depict the intracellular metabolites profiles response to different fermentation conditions, 10 mL of the OBlmT/ManAB fermentation broth from cultures in fermentation medium I and II, respectively, was collected at 48 h, 84 h and 108 h. Each 10 mL sample was quenched immediately with 40 mL of 60% pre-cooled methanol (− 80 °C, v/v) and centrifuged (6000×*g*, 4 °C) to collect cell pellet, which was then washed three times with 0.9% NaCl solution (4 °C, w/v). Subsequently, the extraction and chemical derivatization of intracellular metabolites were conducted according to the methods published by Xia et al. [25]. Succinic d4 (St. Louis, MO) was added in advance and served as the internal standard for GC–MS analysis. All the derivatized samples were measured using a GC–MS using Agilent 7890B/7000D equipped with an HB-5 capillary column (30 m × 0.25 mm × 0.25 μm, Agilent) for gas chromatography. The parameters for GC–MS determination were set as following: split ratio was 5:1; both of injector temperature and interface temperature were set as 280 °C; the velocity of carrier gas (helium) was 1.0 mL/min. The column temperature program encompassed 70 °C hold for 2 min, followed by a 5 °C/min ramp to 280 °C, where it was held for 3 min. The ionization mode was electron ionization (EI) mass spectra and the energy of ionization was 70 eV, the ionization temperature was 250 °C. The MS scanning range was 50–650 m/z. Both of the mass spectrum extraction and identification of metabolites were performed by Agilent Masshunter Qualitative Analysis B.08.00. The metabolites detected by GC–MS were shown in Additional file 1: Table S3. After the normalization with the internal standard succinic d4 and cell biomass, the relative abundance matrix of the whole identified metabolites at each sampling time point was loaded into SIGMA 13.0 (Umetrics, Umea, Sweden) for Principal Component Analysis (PCA) and Partial Least Squares Analysis (PLS). Five biological replicates were conducted for each sample.

## Results

### Bleomycin fermentation characteristics and morphological changes in *S. verticillus* mediated by GlcNAc

**Fig. 1:**
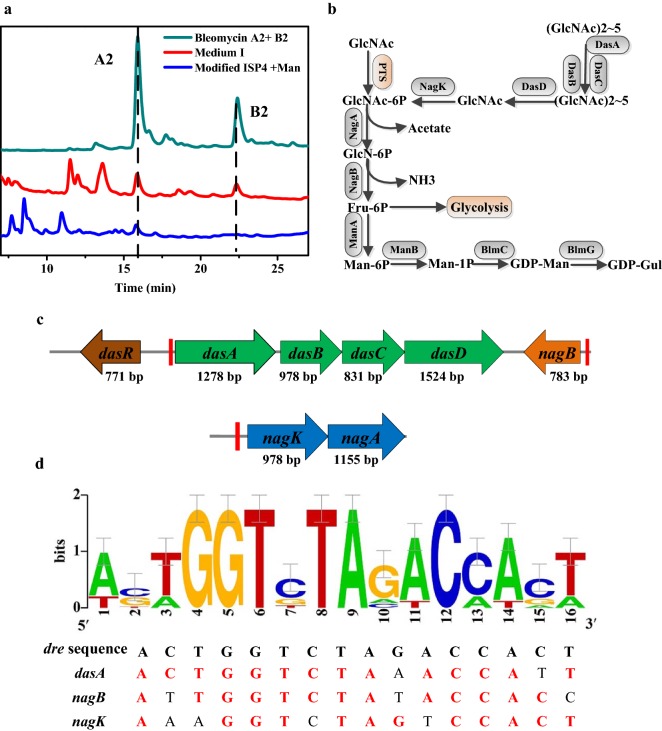
Effect of GlcNAc addition on the fermentation characteristics of *S. verticillus* in chemically defined medium and schematic diagram of GlcNAc metabolism. **a** Bleomycin productions in modified ISP4 with 5 g/L mannose and modified ISP4 medium with 5 g/L GlcNAc (medium I). The HPLC spectrum of standard bleomycins was showed in cyan curve, and the peak times of bleomycin A2 and B2 had been marked by black dotted lines. **b** Metabolic pathway and enzymes in GlcNAc metabolism and GDP-mannose synthesis. **c** Length and organization of the gene sequences of the *dasR*, *dasA*, *dasBCD*, *nagB* and *nagKA* operons; the *dre* elements located in the promote region of *dasA*, *nagB* and *nagK* are indicated in red columns. **d** WebLogo of the *dre* element, and nucleotide sequence of *dre* motifs in *S. verticillus*. The DNA binding sequences of DasR were collected from literatures [10, 42], and were deposited to WebLogo to create sequence logos. The *dre* sequence was proposed by Rigali et al. [10]. Based on MAST analysis, the DNA sequences of the *dre* motif in the promoter region of *dasA*, *nagB* and *nagK* were shown, in which the red font indicated similarity with the *dre* consensus sequence

The yields of bleomycins A2 and B2 produced by *S. verticillus* ATCC 15003 in complex fermentation medium reached 22.18 ± 3.29 mg/L and 38.26 ± 2.36 mg/L, respectively, while none were detected in modified ISP4 medium, even with the addition of 1 g/L tryptone and 0.5 g/L yeast extract. To find the key factors affecting the synthesis of bleomycins, 5 g/L of glucose, mannose, fructose, arabinose, lactose, galactose, chitin, GlcNAc and d-Glucosamine (GlcN) were individually added to the fermentation medium as supplementary carbon sources. Surprisingly, only the addition of 5 g/L mannose in modified ISP4 medium made the yield of bleomycin reach the detection limit of HPLC, while 5 g/L of GlcNAc in modified ISP4 (medium I) significantly increased the production of bleomycin A2 and B2 to 22.91 ± 3.36 mg/L and 11.44 ± 2.39 mg/L, respectively (Fig. 1a). It was predicted that the carbohydrate metabolism might be one of the limiting factors for bleomycins overproduction. In *S. coelicolor*, GlcNAc has been proven to be a signaling molecule related to the regulation of morphological differentiation and the synthesis of antibiotic in response to changes in the nutritional status [10]. Next, spores of *S. verticillus* ATCC 15003 were streaked onto ISP4 and MM solid medium with the addition of different concentrations of GlcNAc (Additional file 1: Fig. S4). GlcNAc concentrations over 5 g/L led to developmental retardation and the aerial hyphae did not form grey-green spores, while colonies growing on ISP4 solid medium containing 25 g/L of GlcNAc exhibited an abnormal, wrinkled phenotype. On the contrary, the addition of 5 g/L GlcNAc in MM could accelerate spore maturation, but aerial mycelia were prevented from developing into spores at high concentrations (25 g/L GlcNAc). Overall, GlcNAc not only improved the production of bleomycins but also induced a series of morphological changes under different nutritional conditions. Consequently, we decided to investigate the effect of GlcNAc on bleomycin synthesis in *S. verticillus* in hopes of obtaining higher bleomycins titers.

### Regulation of GlcNAc metabolism by the global regulator DasR

Since the whole genome sequence of *S. verticillus* ATCC 15003 has not been published, the genes related to GlcNAc metabolism (*dasR*, *dasA*, *dasB*, *dasC*, *dasD*, *nagK*, *nagA* and *nagB*) in *S. verticillus* were identified using SiteFinding PCR (Fig. 1b, c). The amino acid sequence of DasR showed 88.93% and 88.49% identity with its homologs from *S. coelicolor* A3(2) and *S. griseus*, respectively. The *dasR* was located adjacent to *dasABCD* operon, which is involved in the transport and metabolism of *N*,*N*ʹ-diacetylchitobiose in *S. coelicolor* [33]. Similarly, *dasABCD* in *S. verticillus* was also found to be located upstream of *dasR*, and showed 69.88%, 77.37%, 74.64% and 69.12% amino acid sequence identity with their respective homologs in *S. coelicolor* A3(2). Compared with *dasABCD*, a divergently transcribed gene *nagB* possessed higher amino acid identify (80.46%) with its homologs in *S. coelicolor* A3(2). Likely due to the presence of special sequence features, a partial intergenic region between *dasA* and *dasB* in *S. verticillus* could not be resolved successfully by Sanger sequencing. Additionally, *nagK* and *nagA* in *S. verticillus* showed 71.33% and 72.32% identity with SCO4285 (*nagK*) and SCO4284 (*nagA*) in *S. coelicolor* A3(2), respectively. The amino acid sequence alignments indicated that the enzymes involved in GlcNAc catabolism in *S. verticillus* might present considerable functional similarity to those of *S. coelicolor* A3(2).

**Fig. 2:**
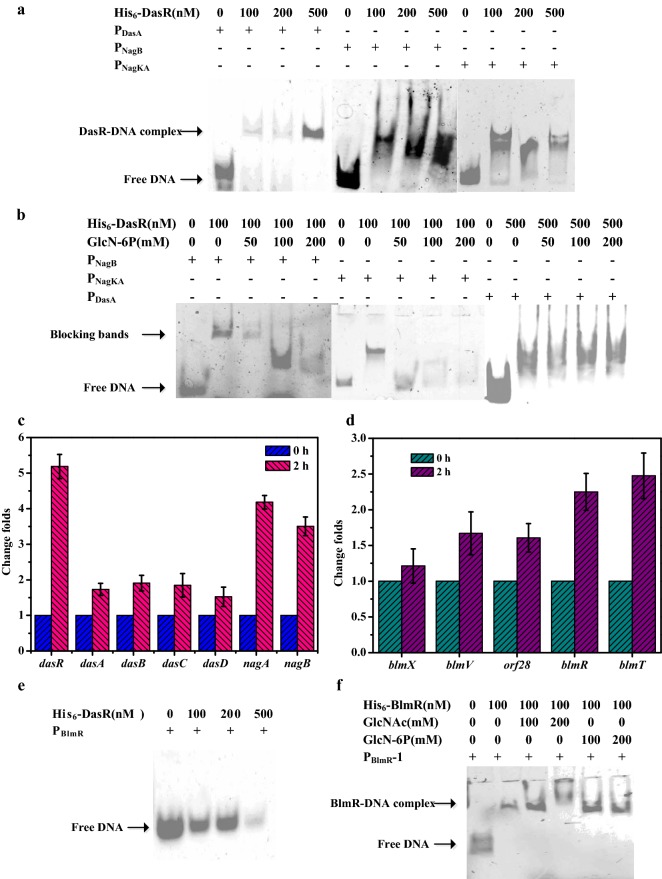
Effect of DasR on the promoter ability of *das* genes, *nag* genes and *blmR*. **a** EMSAs of His_6_-DasR binding to probes P_DasA_, P_NagB_ and P_NagKA_. **b** EMSAs analysis of the effect of GlcN-6P on the binding of DasR to probes P_DasA_, P_NagB_ and P_NagKA_. **c** Change folds of GlcNAc metabolism genes at 0 h and 2 h after GlcNAc induction. **d** Transcription levels of bleomycin biosynthetic gene cluster at 0 h and 2 h after GlcNAc induction. **e** EMSAs displayed the binding of His_6_-DasR to the whole promoter region of gene *blmR*. **f** EMSAs analysis indicating the effect of GlcNAc and GlcN-6P on the BlmR-DNA complex

GlcNAc induction could relieve the repression of DasR on the expression of *nag* and *das* genes in the amino sugar metabolism pathway, as well as the negative effect on pathway-specific regulators in the antibiotic synthesis gene clusters [15]. DasR has been proved to bind to 16 bp DasR-responsive elements (*dre* element) in the promoter regions of its target genes. Moreover, the intermediate products of GlcNAc catabolism, especially GlcN-6P, can induce the dissociation of the DasR-DNA complex by competitively binding to DasR [14, 16]. Here, the known DasR binding sites identified in *Streptomyces* were collected and loaded into WebLogo (http://weblogo.berkeley.edu/logo.cgi) to create a sequence logo (Fig. 1d). Three DasR binding sites in the promoter regions of *dasA*, *nagK* and *nagB*, with high degrees of similarity to the *dre* sequence, were respectively identified in *S. verticillus* using the Motif Alignment & Search Tool (MAST) (Fig. 1d). The probes P_DasA_, P_NagB_ and P_NagKA_ containing the corresponding *dre* sites in the promoter regions of *dasA*, *nagB* and *nagK* were used in EMSAs, and the results showed that DasR indeed had the ability to bind to the promoter regions of *dasA*, *nagB* and *nagK* in *S. verticillus*. As an intracellular metabolite, high concentrations of GlcN-6P have been considered to be lethal. Thus, the effect of the GlcN-6P ligand on the binding of DasR was also detected in vitro by EMSAs. The addition of GlcN-6P (over 100 mM) into the EMSAs mixture resulted in the loss of the shifted bands corresponding to the DasR-P_NagB_ complex and the occurrence of a new shifted band, while over 50 mM of GlcN-6P would lead to the dissociation of the DasR-P_NagKA_ complex but had no obvious impact on the DasR-P_DasA_ complex (Fig. 2a, b).

To further verify whether the addition of GlcNAc in the liquid medium relieved the inhibition of amino sugar metabolism by DasR, the fermentation characteristics of *S. verticillus* ATCC 15003 after GlcNAc addition at different times were first investigated. The results indicated that the addition of GlcNAc during 0–48 h resulted in a similar bleomycin yield (Additional file 1: Fig. S5). Here, GlcNAc was added to the modified ISP4 medium after the cultivation of 36 h, at which time the strain was in the early exponential phase. Samples for RT-qPCR analysis were collected at 0 h and 2 h after GlcNAc induction. Genes with relative expression exceeding 2 or below 0.5 were considered significantly differentially expressed in this study. As shown in Fig. 2c, the expression levels of *dasA, dasB, dasC* and *dasD* did not change obviously, but that of *dasR* was increased at 2 h after induction with GlcNAc. Genes in the GlcNAc catabolism pathway include *nagK*, *nagA* and *nagB*, with *nagK* and *nagA* sharing a single transcriptional unit (Fig. 1b, c). The elevated expression levels of *nagA* and *nagB* at 2 h indicated that GlcNAc induction inhibited the binding of DasR to the *dre* sites in the promoter regions of the *nagB, nagK* and *nagA* genes.

### Effect of GlcNAc metabolism on bleomycin biosynthesis

Gene *blmR*, *orf28*, *blmX* and *blmV* are the first genes in operons I, II, III and IV of the bleomycin gene cluster, respectively. The RT-qPCR results indicated that only the expression level of *blmR* was significantly increased, while those of *orf28*, *blmX* and *blmV* were not affected in the presence of GlcNAc (Fig. 2d). As an autoregulator, BlmR is previously characterized as a transcriptional repressor of bleomycin production that binds to a 12-2-12 bp DNA fragment in its own promoter to inhibit the expression of the transcription unit composed of *blmR* and *blmT* [4]. According to gene annotation in NCBI, gene *blmT* encodes a putative transporter with a conserved sulfate transporter and anti-sigma factor antagonist domain. The up-regulation of the transcription level of *blmR* had no direct inhibitory effect on the expression of *blmT*, which increased nearly 2.5 times after GlcNAc induction (Fig. 2c). No *dre* sites were found in the promoter of the *blmR*-*blmT* operon, and DasR could not bind to P_BlmR_ in vitro, which was verified by EMSAs (Fig. 2e). It was suggested that the change in the transcription level of the *blmR*-*blmT* operon was not caused by the disassociation of the DasR-*dre* complex. Whether the GlcNAc metabolites directly affect the binding of BlmR to its target 12-2-12 bp DNA sequence (P_BlmR_-1) was therefore an open question. The result from EMSAs showed that neither GlcNAc nor GlcN-6P could induce the dissociation of the BlmR-DNA complex (Fig. 2f). Due to the positive role of the elevated expression of BlmT in bleomycin biosynthesis, the strain OBlmT was constructed, and the titers of bleomycin A2 and B2 were respectively increased to 32.72 ± 1.83 mg/L and 20.18 ± 3.09 mg/L in the presence of GlcNAc, which were close to those of the BlmR deletion and BlmT overexpression strain DBlmT-ΔBlmR. In addition, the titers of bleomycin A2 and B2 of the BlmR deletion strain DBlmR in modified ISP4 medium without GlcNAc addition were respectively 18.04 and 11.92 mg/L, which were lower than with the addition of GlcNAc (23.88 and 14.82, respectively). It could be inferred that the global regulatory effect of GlcNAc on bleomycin synthesis was not limited to the relief of negative regulation of BlmT by the pathway-specific regulator BlmR, but was related to the other factors as well. Nevertheless, genetic engineering of *blmT* under GlcNAc induction offered a feasible method for increasing the bleomycin yield.

### Improved GDP-mannose supply to increase bleomycin yield under GlcNAc induction

**Fig. 3:**
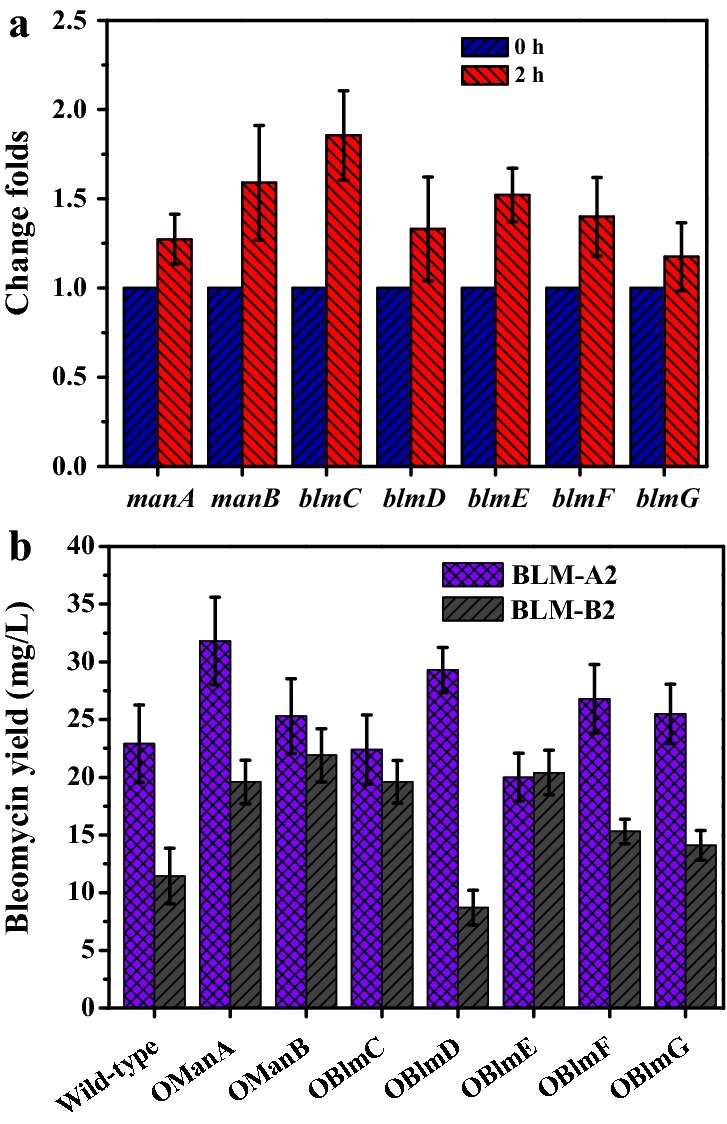
**a** Effect of GlcNAc induction on the expression of genes related to GDP-mannose synthesis and glycosylation. **b** Effects of the overexpression of genes in mannose catabolism on the production of bleomycin A2 and B2. The data were the average values from three repeated fermentation trials

According to the pathway analysis of amino sugar metabolism, GlcNAc can be converted to fructose-6-phosphate (frucose-6P) catalyzed by NagA and NagB. Improved transcription levels of *nagA* and *nagB* under GlcNAc addition indicated that GlcNAc might be assimilated efficiently by *S. verticillus*. Fructose-6P is not only the end-product of GlcNAc metabolism, but it can also be converted into GDP-mannose and GDP-gulose in bleomycin biosynthesis, catalyzed by ManA, ManB, the sugar synthase BlmC and the sugar epimerase BlmG (Fig. 1b). GDP-mannose and GDP-gulose are the direct precursors of the disaccharide unit that are attached to the bleomycin aglycone through glycosylation catalyzed by the sugar transferase BlmE, hydroxylase BlmF and carbamoyltransferase BlmD [32]. Whether the increased bleomycins yield under GlcNAc induction could be associated with enhanced biosynthesis of GDP-mannose, the DNA fragments of *manA* and *manB* from *S. verticillus* were sequenced by SiteFinding PCR, and the corresponding amino acid sequences exhibited 69.10% and 82.09% identity with SCO3025 (*manA*) and SCO3028 (*manB*) from *S. coelicolor* A3(2), respectively. RT-qPCR was utilized to measure the expression of genes involved in disaccharide unit synthesis (*manA*, *manB*, *blmC* and *blmG*) and glycosylation (*blmE*, *blmF* and *blmD*) in the presence of GlcNAc. The results (Fig. 3a) indicated that the expression of genes listed above was not obviously changed (change folds below 2). To improve the specific sugar precursor supply under GlcNAc induction, gene *manA*, *manB*, *blmC*, *blmD*, *blmE*, *blmF* and *blmG* related to bleomycins glycosylation were overexpressed in strains OManA, OManB, OBlmC, OBlmD, OBlmE, OBlmF and OBlmG, respectively. The fermentation results in Fig. 3b showed that the total production of bleomycin A2 and B2 was higher in OManA and OManB than in the other strains, with bleomycin A2 and B2 titers reaching 31.80 ± 1.93 mg/L and 19.63 ± 2.84 mg/L in OManA and 25.3 ± 1.42 mg/L and 21.9 ± 2.05 mg/L in OManB, respectively. These results indicated that ManA and ManB might catalyze rate-limiting steps that were critical for increasing bleomycin production. For the improvement of bleomycin yield under GlcNAc induction, the tandem overexpression strain OBlmT/ManAB was constructed, and its corresponding titers of bleomycin A2 and B2 in medium I were increased to 40.37 ± 2.04 mg/L and 23.51 ± 3.18 mg/L, respectively.

### Identification of key modules related to bleomycin overproduction based on different GlcNAc-derived media

**Fig. 4:**
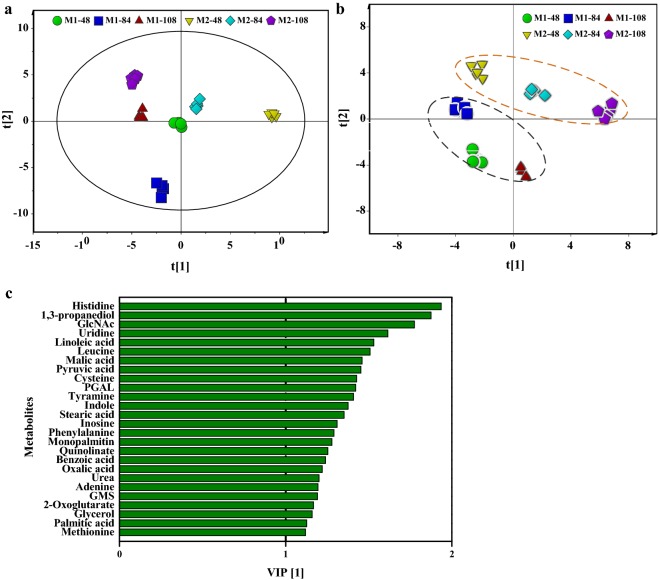
Statistical analysis of intracellular metabolites data measured by GC–MS. Samples were collected at 48 h, 84 h and 108 h for a comparison between medium I and medium II. **a** PCA- derived loading plots. **b** PLS-derived loading plots. Samples from medium I and medium II were inside the black and orange dashed ellipses, respectively. **c** PLS-derived VIP plots with 26 metabolites with VIP values exceeding 1. GlcNAc, *N*-acetylglucosamine; PGAL, glyceraldehydes 3-phosphate; GMS, glycerol monostearate

In addition to the effect of GlcNAc metabolism on transcription factors, a previous study indicated that GlcNAc assimilation could lead to changes in the concentrations of various intracellular nitro-containing metabolites (especially amino acids, purines, and pyrimidines) [34]. On the basis of medium I, the optimization of the fermentation medium for the production of bleomycins was performed using OBlmT/ManAB as bleomycins-producing strain. The obtained medium II contained high levels of chitin-derived substrates including chitosan and GlcNAc, which increased the titers of bleomycin A2 and B2 to 52.16 ± 1.93 mg/L and 30.02 ± 3.04 mg/L, respectively. To investigate the key factors limiting the yield of bleomycins in GlcNAc-derived media, the intracellular metabolic profiles of strain OBlmT/ManAB in medium I and medium II were assessed by comparative metabolomics. A total of 92 metabolites were detected, 72 of which were identified by GC–MS (Additional file 1: Table S3). Subsequently, PCA was utilized to analyze the differences between the samples. From the PCA-derived score plot (R2X(cum) = 0.889, Q2(cum) = 0.809) in Fig. 4a, five parallel samples taken at the same time point were tightly clustered, and six samples were clearly separated using Hotelling’s T^2^ test (95%), both of these results reflected different intracellular metabolic characteristics in the selected fermentation phases. To connect the changes of metabolite fluxes to bleomycins synthesis, PLS analysis of all the samples was carried out using metabolites as X variables and bleomycin yield as the Y variable. In the PLS-derived score plot (R2X(cum) = 0.745, R2Y(cum) = 0.934, Q2(cum) = 0.897) shown in Fig. 4b, the samples from different media were distributed in different quadrants, with samples from medium I being found in lower regions of quadrants than those from medium II. Simultaneously, the variable importance of the projection (VIP) plots of each metabolite was generated, where a higher VIP value (exceeding 1) indicated a more significant contribution to the differences in bleomycin production. According to the VIP scores (Additional file 1: Table S3) and KEGG pathway analysis, 30 chemical substances were identified as key metabolites related with bleomycins overproduction, most of which were involved in the metabolism of amino acids, long-chain fatty acids, pyruvate and the TCA cycle. The bleomycins aglycone is formed by the dehydration condensation of 9 amino acids, including one l-serine, two l-asparagines, one l-histidine, one l-alanine, one l-threonine, one β-alanine and two l-cysteines [2]. Together, the biomarkers of amino acids metabolism can therefore be expected to play critical roles in the metabolic flux distribution and the supply of various amino acid precursors between chitin-derived media.

### Further improvement of bleomycin yield by metabolic profiling analysis

**Fig. 5:**
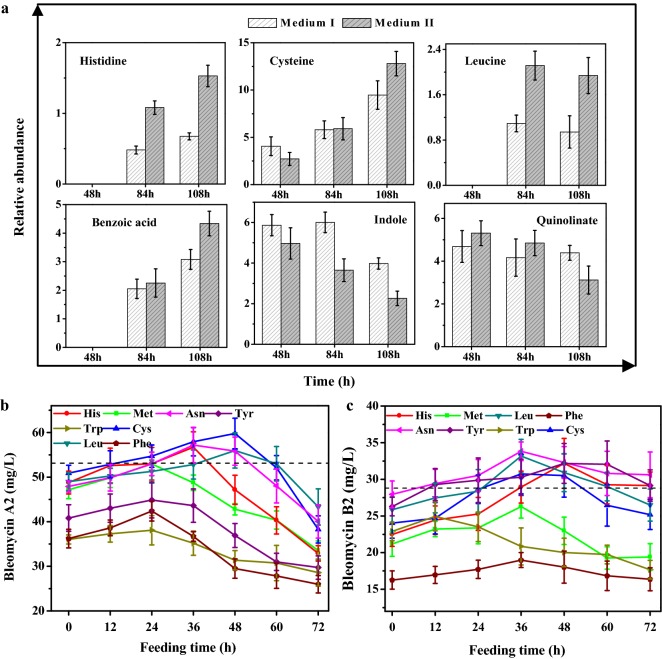
**a** Comparative analysis of biomarkers associated with bleomycin production between medium I and medium II at 48 h, 84 h and 108 h. **b**, **c** Effects of exogenous addition of key amino acids in medium II on bleomycin A2 (**b**) and B2 (**c**) production at various times. The black dashed lines represented the productions of bleomycin A2 and B2 in medium II, which was used as the control

As seen in Fig. 4c, 9 metabolites associated with amino acid metabolism were identified as biomarkers related to bleomycin biosynthesis. Those were histidine, leucine, cysteine, tyramine, indole, phenylalanine, quinolinate, benzoic acid and methionine. Among them, histidine and cysteine are direct precursors of bleomycins, while methionine can be converted into S-adenosylmethionine (SAM) to provide methyl groups for bleomycins synthesis. The changes of relative abundance of these biomarkers are shown in Fig. 5a. Compared with medium I, the relative abundance of histidine increased with time and was always higher in medium II, while the relative abundance of cysteine in medium II was noticeably increased only at 108 h. These results indicated that the flux of histidine and cysteine was enhanced in the later phase of bleomycins fermentation. Surprisingly, leucine was also identified as a biomarker, although it does not directly contribute to the supply of precursors for bleomycins synthesis. However, leucine can be converted into acetyl CoA, which can be converted into the bleomycins precursor malonyl-CoA by acetyl-CoA carboxylases [35], and the relative abundance of leucine in medium II was obviously higher than that in medium I. Phenylalanine, tyramine, indole, quinolinate and benzoic acid were associated with aromatic amino acids metabolism according to KEGG pathway analysis. In medium II, only the flux of benzoic acid was increased, while phenylalanine and tyramine were not detected and the relative abundance of indole and quinolinate gradually decreased. It can be inferred that the weakening of aromatic amino acids pathways appears to affect bleomycin synthesis although none of them are the direct precursors.

To verify the deduced regulation points in bleomycins synthesis, a rational feeding experiment was conducted based on the above-described predictions. 1.5 g/L histidine, leucine, cysteine, methionine, tryptophan, tyrosine and phenylalanine were added into the medium II at different fermentation phases, respectively. As seen in Fig. 5b, c, the productions of bleomycin A2 and B2 increased after the addition of histidine (36 h and 48 h), leucine (48 h) and cysteine (48 h and 36 h) in medium II, respectively. Among the aromatic amino acids, only tyrosine (48–60 h) enhanced the production of bleomycin B2, while exogenous feeding of either tryptophan or phenylalanine had the negative effect on the bleomycin yields, which indicated that the down-regulation of the flux of aromatic amino acids during fermentation would be beneficial for the accumulation of bleomycins. Although SAM is the precursor for bleomycin biosynthesis, the addition of methionine did not significantly promote the synthesis of bleomycins. Among the 9 amino acid precursors of the bleomycins aglycone, asparagine was the only one that was not detected in either medium. The fermentation with the addition of 1.5 g/L asparagine in medium II indicated that asparagine played an active role in bleomycins production based on the results of feeding at 36 h. To further improve the bleomycins production, the combined feeding of asparagine (36 h), histidine (36 h), leucine (48 h), and cysteine (36 h) in chitin-derived medium II was tested, and was found to increase the production of bleomycin A2 and B2 to 61.79 ± 3.17 mg/L and 36.9 ± 2.33 mg/L, respectively.

## Discussion

The synthesis of antibiotics in *Streptomyces* is influenced by multiple factors, such as primary metabolism, transcriptional regulation, nutritional status and morphological development [8]. To explore the limiting factors during the synthesis of bleomycins, the optimization of the fermentation medium was first conducted in modified ISP4 liquid fermentation medium, in which the strain was initially unable to synthesize bleomycins. Mannose is the direct precursor of GDP-mannose and GDP-gulose, which together constitute the disaccharide structure of bleomycins [36]. The addition of mannose in modified ISP4 liquid medium was able to improve the bleomycins production to the detection limit of HPLC, while a higher yield was achieved in the case of GlcNAc addition. In *S. coelicolor* A3(2), the high concentration of GlcNAc blocks spore development and antibiotic production under nutrient-rich condition (R2YE solid medium), but it accelerates the sporulation and synthesis of the antibiotics undecylprodigiosin and actinorhodin under nutrient-limited conditions (MM medium) [10]. Indeed, GlcNAc was found to inhibit the spore maturation of *S. verticillus* in modified ISP4 solid medium, but the production of bleomycins A2 and B2 was improved following the addition of GlcNAc in modified ISP4 liquid medium. It could be concluded that the effect of GlcNAc metabolism on bleomycins production in *S. verticillus* might be different from its effect on the induction of antibiotic synthesis in *S. coelicolor* under nutrient-limited conditions.

Several lines of evidence indicate that GlcNAc plays a vital role in the control of morphological differentiation and the onset of antibiotic synthesis based on the nutritional status in the environment [11]. The GntR family repressor DasR is widely considered as a master regulator that binds to specific DNA sequences in the promoter regions of genes involved in chitin/GlcNAc catabolism, sugar hydrolysis, antibiotic production and developmental regulation. However, the intermediate products of GlcNAc catabolism can induce the release of DasR from the target sites, and thereby trigger spore development and antibiotic synthesis [37]. In fact, a number of EMSAs studies have proven that DasR binds to a 16 bp *dre* element and this binding is relieved by the GlcNAc catabolites GlcNAc-6P or GlcN-6P [16]. The prediction of *dre* elements in *Streptomyces* is considered as a potential strategy for identifying the physiological activities that are modulated by GlcNAc [12]. Three *dre* sequence motifs in *S. verticillus* were identified in the promoter regions of *dasA*, *nagK* and *nagB*, respectively. According to the EMSAs results, the promoter activities of *dasA*, *nagKA* and *nagB* were indeed repressed by DasR. However, high concentration of GlcN-6P could alter the binding mode of DasR-P_NagB_ and a new shifted band appeared, indicating that DasR from *S. verticillus* also possessed a similar mode of allosteric modification to its orthologs NagR and DasR^Serry^, in which the DNA-binding domain of NagR had been verified to be re-oriented upon ligand binding [38, 39]. In this study, GlcNAc induction not only relieved the inhibition of the expression of genes related to GlcNAc catabolism by DasR, but also overcame the effect of the increased expression of DasR. This phenomenon has been perviously observed in the wild-type strain *S. coelicolor* A3(2) in the presence of chitin (polymer of GlcNAc) [13]. The stable expression of *dasABCD* was consistent with the idea that the expression of *dasABCD* controlled by DasR was induced by (GlcNAc)_2~5_ metabolism and not by GlcNAc metabolism [40].

There was no doubt that the enhancement of GlcNAc metabolism in *S. verticillus* was ascribed to the removal of the inhibition by DasR. It has been reported that the biosynthesis of most antibiotics in *S. coelicolor* is initiated under GlcNAc induction in poor conditions, which is in fact achieved by impeding the binding of DasR to *dre* element in the promoter region of pathway-specific regulators [14, 16]. Among them, DmdR1 is the only known repressor, which is responsible for the biosynthesis of the siderophores coelichelin and desferrioxamine. DasR could reduce the transcription of gene *dmdR1* by binding to the *dre* element located in its promoter region, which improves the production of siderophores. Under the induction with GlcNAc, the expression of DmdR1 is initiated, which in turn represses the biosynthesis of siderophores [12]. In the bleomycin biosynthesis gene cluster, BlmR acts as an ArsR family repressor that binds to its own promoter to attenuate the transcription of the *blmR*-*blmT* operon, and the combination of the deletion of *blmR* and overexpression of *blmT* could remarkably increase the bleomycin yield [4]. Whether DasR controls the transcription of BlmR through a similar regulation mechanism to that of *dmdR1*, no *dre* element was found and EMSAs indicated that DasR could not bind to the promoter region of BlmR in vitro. In addition to the *dre* element, the binding of DasR to non-canonical sites is also possible, but it requires the cooperative interaction with other cofactors or changes of the DNA conformation, which cannot be observed from in vitro experiment such as EMSAs [15]. Whether DasR affects the regulatory role of the repressor BlmR by binding to the non-canonical sites is a subject of ongoing research in our lab. In addition, GlcNAc and its various polymers, which are important signaling molecules in *Streptomyces*, are assimilated through several complex systems such as PTS, DasABCD and NgcEFG, to cope with the shifting nutrients conditions [11]. So far, the precise roles of these transporters in nutrient sensing and the onset of development are not been well understood.

As a member of the ArsR/SmtB family regulators, BlmR has been verified as an autoregulator that represses the expression of the transcription unit *blmR*-*blmT* by binding to its own promoter region [4]. Under the induction with GlcNAc, the increased expression levels of *blmR* and *blmT* indicated that the binding of BlmR to its target DNA site might be changed or dissociated, leading to the relief of the negative regulation by BlmR. In fact, the binding of most ArsR/SmtB family transcriptional regulators to their target DNA sites is allosterically regulated under the control of various physiological factors such as metal ions or oxidative signals [41]. However, the binding of BlmR could not be affected by metal ions and oxidative signals [4]. Our study also indicated that both GlcNAc and GlcN-6P could not act as ligands to induce the dissociation of the BlmR-DNA complex. GlcNAc is an important signaling molecule related to morphological differentiation in *Streptomyces*, where it can induce changes of global metabolites and regulatory networks. The dynamic transcriptional regulation mechanism of the repressor BlmR triggered by GlcNAc might be linked to the complex regulatory cascades or metabolite spectrum. Nevertheless, it was clear that GlcNAc could improve the bleomycins yield by relieving the transcriptional repression of the *blmR*-*blmT* operon, and the enhancement of BlmT expression under GlcNAc induction could offer a practical approach for the improvement of bleomycin production.

According to the KEGG database, the end-product of GlcNAc metabolism, fructose-6P, can be converted to mannose-1P which is the direct precursor of the disaccharide moiety of bleomycins. The results of RT-qPCR indicated that the transcription of genes involved in GDP-mannose synthesis and bleomycin glycosylation did not change obviously even when GlcNAc catabolism was enhanced. Consequently, the *manA*, *manB*, *blmC*, *blmD*, *blmE*, *blmF* and *blmG* genes were individually overexpressed to determine the limiting step in the specific sugar precursor supply for bleomycins synthesis. Fermentation experiments suggested that bleomycin yields of OManA and OManB were higher than other strains, which suggested that ManA and ManB were the key enzymes controlling the flow of carbon toward the bleomycins disaccharide structure. Previous study found that the enzyme activity of ManA in *S. coelicolor* was lower than that in other organisms [42], and the carbon flux toward mannose catabolism might hence be relatively elevated by the overexpression of ManA. ManB is a bifunctional enzyme with phosphomannomutase and phosphoglucomutase activities, but it is likely to give priority to the GDP-mannose synthesis pathway in *S. coelicolor* [43]. The high sequence identity (82.08%) of ManB from *S. verticillus* to its homolog from *S. coelicolor* might also indicate their large functional similarity. Under GlcNAc induction, the combined overexpression of *blmT*, *manA* and *manB* led to the increase of the production of bleomycin A2 and B2 in medium I to 40.37 mg/L and 23.51 mg/L, respectively.

**Fig. 6:**
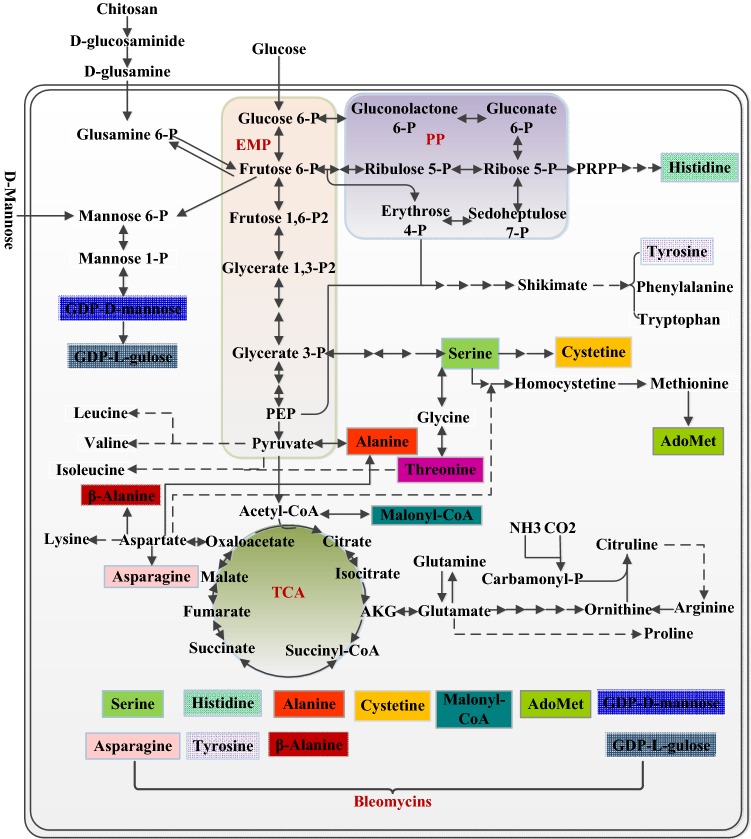
Schematic view of the metabolic pathway associated with bleomycin synthesis in *S. verticillus*. The precursors for bleomycin production were represented by colored rectangular boxes. The dashed arrows indicated that there were multiple reaction steps in this line. EMP, Embden–Meyerhof–Parnas pathway; PP, Pentose Phosphate pathway; PEP, Phosphoenolpyruvate; PRPP, 5-Phosphoribosyl 1-Pyrophosphate; TCA, Tricarboxylic acid cycle; AKG, α-Ketoglutaric acid

Bleomycin aglycone is directly come from eight natural amino acids and one unnatural amino acid β-alanine (Fig. 6). Moreover, GlcNAc assimilation has the positive effect on the abundance of intracellular amino acid pools [34]. To explore the limiting metabolites related to bleomycin biosynthesis in GlcNAc-derived media, the intracellular metabolite profiling was investigated by comparative metabolomics in this study. Here, histidine, cysteine, leucine, aromatic amino acids and methionine were considered to present a high correlation with bleomycins overproduction according to PLS analysis. It is known that methionine can be catalyzed to form SAM, which is an important methyl donor for antibiotic synthesis and DNA methylation [44]. There are two methylation reactions with the participation of SAM during the bleomycins synthesis, but the addition of methionine in medium II could not improve the production of bleomycins. A similar lack of a promoting effect was also reported in the synthesis of antibiotics carrying methyl or methoxy groups, which might result from the repression of methyltransferases by methionine [45]. As the direct precursors of the bleomycins aglycone, the addition of either histidine or cysteine could increase the bleomycin yield. Furthermore, an elevated bleomycins yield was also obtained via the addition of leucine. It has been proven that the addition of leucine can lead to the increase of intracellular pool of acetyl-CoA, which in turn could be easily transformed into malonyl-CoA through a one step enzymatic reaction [25]. Based on the stereochemical structure of bleomycins, malonyl-CoA is the sole substrate used to assemble to the skeleton of the bleomycins aglycone by the specific hybrid NRPS-PKS synthases [46]. With the exception of tyrosine, the feeding of aromatic amino acids was not conducive to the increase of bleomycin yield. According to the changes of relative abundances of metabolites in aromatic amino acids pathway, it could be suggested that the intracellular metabolic flux flowing to aromatic amino acids might tend to decline under bleomycins overproduction condition. Although asparagine was not detected in the fermentation process, its positive effect on the enhancement of bleomycins was verified by feeding assay. Finally, the combined feeding of the four amino acids asparagine, leucine, histidine and cysteine in medium II further increased the production of bleomycins, illustrating the applicability of metabolomics in microbial bio-manufacturing.

## Conclusions

GlcNAc is a global signaling molecule and was demonstrated in this study to significantly initiate the biosynthesis of bleomycins. To explore the effects of GlcNAc on bleomycin biosynthesis, the *das* and *nag* genes involved in GlcNAc regulation and metabolism in *S. verticillus* were investigated, and the results showed that the repression of the expression of *nagB* and *nagKA* by the global transcription factor DasR was relieved under the induction with GlcNAc. Although no direct evidence proved that the expression of BlmR was regulated by DasR, the negative effect of BlmR on its target gene *blmT* was indeed relieved under GlcNAc induction, which was the main cause of elevated bleomycin production and offered an effective strategy for strain improvement. By applying this strategy in combination with the key rate-limiting enzymes ManA and ManB in bleomycins glycosylation, the overproduction strain OBlmT/ManAB was constructed. Assisted by metabolic profiling analysis, the production of bleomycin A2 and B2 in GlcNAc-containing medium was ultimately increased to 61.79 and 36.9 mg/L, respectively, and the product ratio of 2:1 was close to the commercial bleomycin-based drug formulation. Here, signal molecule GlcNAc regulation and assisted metabolic profile analysis effectively enhanced the bleomycin yield. Moreover, the utilization of various chitin-derived constituents in medium not only improved the applicability and economic feasibility of the fermentation process for industrial bleomycin production, but also offered an example for the other improving the bio-production processes of other antibiotics.

## Supplementary information

**Additional file 1: Table S1.** strains and plasmids used in this study. **Table S2.** Primers used in this study. **Table S3.** List of intracellular metabolites detected by GC–MS. **Figure S1.** Detail schematic diagram of chromosome walking by SiteFinding PCR and nested PCR. **Figure S2.** SDS-PAGE profile of 6 × His-DasR used in EMSA assay. **Figure S3.** DNA sequence of probes used in EMSAs. **Figure S4.** Effect of GlcNAc addition on spore morphogenesis in different mediums. **Figure S5.** Fermentation characteristics of bleomycins in the different GlcNAc addition times. The control group was without GlcNAc.

## Data Availability

All data generated and analyzed during this study are included in this manuscript and in its Additional file 1.

## Abbreviations

GlcNAc: *N*-acetylglucosamine
RT-qPCR: Real time fluorescence quantitative PCR
EMSAs: Electrophoretic mobility shift assays
ManA: Phosphomannose isomerase
ManB: Phosphomannomutase
PTS: Phosphoglucosyltransferase system
GlcNAc-6P: *N*-acetylglucosamine 6-phosphate
GlcN-6P: Glucosamine-6-phosphate
ATCC: American type culture collection
MOPS: 3-Morpholinopropanesulfonic acid
LB: Luria–Bertani medium
HPLC: High performance liquid chromatography
TSB: Tryptic Soy Broth medium
MM: Minimal medium
NCBI: National Center for Biotechnology Information
GSP: Gene specific primer
IPTG: Iso-propyl-β-d-thiogalactoside
GC–MS: Gas chromatograph mass spectroscopy
EI: Electron ionization
PCA: Principal component analysis
PLS: Partial least squares analysis
GlcN: d-Glucosamine
*dre*: DasR-responsive elements
MAST: Motif alignment and search tool
VIP: Variable importance of the projection
SAM: S-adenosylmethionine
