# Supplementary material for: Enhancement of bleomycin production in Streptomyces verticillus through global metabolic regulation of N-acetylglucosamine and assisted metabolic profiling analysis
Source: Microb Cell Fact. 2020 Feb 13;19:32. doi: 10.1186/s12934-020-01301-8 (PMC7017467; doi:10.1186/s12934-020-01301-8)
Supplement: Supplementary file 1 — Additional file 1: Table S1. strains and plasmids used in this study. Table S2. Primers used in this study. Table S3. List of intracellular metabolites detected by GC–MS. Figure S1. Detail schematic diagram of chromosome walking by SiteFinding PCR and nested PCR. Figure S2. SDS-PAGE profile of 6 × His-DasR used in EMSA assay. Figure S3. DNA sequence of probes used in EMSAs. Figure S4. Effect of GlcNAc addition on spore morphogenesis in different mediums. Figure S5. Fermentation characteristics of bleomycins in the different GlcNAc addition times. The control group was without GlcNAc. [file 12934_2020_1301_MOESM1_ESM.pdf]

Additional file 1

Microbial Cell Factories

Enhancement of bleomycin production in *Streptomyces verticillus* through global metabolic regulation of N-acetylglucosamine and assisted metabolic profiling analysis

Hong Chen[1,2,#], Jiaqi Cui[1,2,#], Pan Wang[1,2], Xin Wang[1,2], Jianping Wen[1,2]*

[1] Key Laboratory of Systems Bioengineering (Ministry of Education), Tianjin University, Tianjin 300072, P. R. China

[2] SynBio Research Platform, Collaborative Innovation Center of Chemical Science and Engineering (Tianjin), School of Chemical Engineering and Technology, Tianjin University, Tianjin 300072, P. R. China

[#] These authors contributed equally to this work

*Correspondence author: Jianping Wen

Telephone: +86-022-27892061;

Fax: +86-022-27892061;

E-mail: jpwen@tju.edu.cn

Table S1 strains and plasmids used in this study

Strains or plasmids	Description	Source or reference
**Strains**		
Streptomyces verticillus ATCC15003	Wild-type	American Type Culture Collection, USA
DBlmR	*blmR* deletion strain based on wild strain	In our laboratory
DBlmT-ΔBlmR	blmT overespression strain based on DBlmR	In our laboratory
OManA	*manA* overexpression strain based on wild strain	This study
OManB	*manB* overexpression strain based on wild strain	This study
OBlmC	*blmC* overexpression strain based on wild strain	This study
OBlmD	*blmD* overexpression strain based on wild strain	This study
OBlmE	*blmE* overexpression strain based on wild strain	This study
OBlmF	*blmF* overexpression strain based on wild strain	This study
OBlmG	*blmG* overexpression strain based on wild strain	This study
OManAB	*manA* and *manB* double overexpression strain based on wild strain	This study
OBlmT/ManAB	*blmT, manA and manB* co-expression strain based on wild strain	This study
E. coli		
DH5α	Cloning host for plasmid construction	Novagen, USA
S17-1	Methylation proficient donor strain for *E. coli-S. verticillus* conjugation	Novagen, USA

E. coli BL21(DE3)	Host for LacZ reporter asssay	Novagen, USA
**Plasmids**		
pUC18	Vector for Site-Finding PCR and clone	Novagen, USA
pIB139	pSET152-derived vector containing the promoter ermEp*	Chen *et al* [1]
pET28a(+)	Expression vector	Novagen, USA
pLacZ	LacZ reporter plasmid	Chen *et al* [1]
pET28a(+)/BlmR	pET28a(+)-derived vector used for the expression of BlmR	Chen *et al* [1]
pET28a(+)/DasR	pET28a(+)-derived vector used for the expression of DasR	This study
pLacZ/P_{DasA},	pLacZ-derived vector in which gene *lacZ* was under the control of the promoter of DasA	This study
pLacZ/P_{NagB}	pLacZ-derived vector in which gene *lacZ* was under the control of the promoter of NagB	This study
pLacZ/P_{BlmR}	pLacZ-derived vector in which gene *lacZ* was under the control of the promoter of BlmR	This study
pIB139/*manA*	pIB139 containing *manA* gene	This study
pIB139/*manB*	pIB139 containing *manB* gene	This study
pIB139/*blmC*	pIB139 containing *blmC* gene	This study
pIB139/ *blmD*	pIB139 containing *blmD* gene	This study
pIB139/ *blmE*	pIB139 containing *blmE* gene	This study
pIB139/ *blmF*	pIB139 containing *blmF* gene	This study
pIB139/ *blmG*	pIB139 containing *blmG* gene	This study
pIB139/*manA*/*manB*	pIB139 containing *manA* and *manB* gene	This study

Table S2 Primers used in this study

Primers	DNA sequence (5'-3')	Use (s)
**Primers used for cloning of conserved sequence**		
PCF/*dasR*	TGCACTGCAGTSGCSGCSGARTTCGACACCT	Amplification of the conserved sequence in *dasR*
PCR/*dasR*	CTAGTCTAGAAKSAGCATSGGSAGSCCCACG	
PCF/*nagA*	TGCACTGCAGCACTGGATCGTCCCCGGCTTCGT	Amplification of the conserved sequence in *nagA*
PCR/*nagA*	CTAGTCTAGAGAGCCSGCGATCGARCCSG	
PCF/*manA*	TGCACTGCAGTACGCCTGGGGCTCCACCACCGC	Amplification of the conserved sequence in *manA*
PCF/*manA*	CTAGTCTAGATSAGBCCGCASCGCAGCACGT	
PCF/*manB*	TGCACTGCAGTGTTCACSGCCWSSCACAA	Amplification of the conserved sequence in *manB*
PCF/*manB*	CTAGTCTAGAKYGCCGCCGYRCTCSYKGAC	
PCF/*16sRNA*	TGCACTGCAGGCCCGCGGCCTATCAGCTTGTTG	Amplification of the conserved sequence in *16sRNA*
PCF/*16sRNA*	CTAGTCTAGACGGGCTTTCACATCCGACGCG	
**Primers for degenerate PCR**		
SiteFinder	CACGACACGCTACTCAACACACCACCTCGCACAGCGTCCTCAAtctagaNNNCGGGCGC	
SFP1	CACGACACGCTACTCAACAC	
SFP2	ACTCAACACACCACCTCGCACAGC	
**Primers for gene overexpression**		
OF/blmC	ggttggtaggatccaGTGAACACCGACCTGCCCCG	Amplification of BlmC encoding sequence for OBlmC construction
OR/blmC	cgcggccgcggatccTCATGGGGTGTCTCCCTCGC	

OF/blmD	ggttggtaggatccaTCAGGAGTACCCCATGAGC	Amplification of BlmD encoding sequence for
OR/blmD	cgcggccgcggatccTCATGAGCGGGCCGCCGT	OBlmD construction
OF/blmE	ggttggtaggatccaCACCGAAGGGAGGGACCCCA	Amplification of BlmE encoding sequence for
OR/blmE	cgcggccgcggatccTCTAGATCGGCGCTCATGGGGTACTC	OBlmE construction
OF/blmF	ggttggtaggatccaGTGAAGGACCTCGGCCGGCT	Amplification of BlmF encoding sequence for
OR/blmF	cgcggccgcggatccTGCCGCACACGGGCCTCACT	OBlmF construction
OF/blmG	ggttggtaggatccaGTGACATGGACCGTGGTGACC	Amplification of BlmG encoding sequence for
OR/blmG	cgcggccgcggatccTGCGGTGCTCAGGCATCGGC	OBlmG construction
OF/manA	ggttggtaggatccaTGGACCGCCTCGCCAACAC	Amplification of ManA encoding sequence for
OR/manA	cgcggccgcggatccGAACCGCCTTCAGACGGCGA	OManA construction
OF/manB	ggttggtaggatccaGTGGCTGATCTGCGCAGCT	Amplification of ManB encoding sequence for
OR/manB	cgcggccgcggatccTTCAAACCGTCGAGGGC	OManB construction
OF/blmT	ggttggtaggatccaGTGAGCTCCCTCGCCGTCC	Amplification of BlmT encoding sequence for
OR/blmB	cgcggccgcggatccTCCTCATCGTCGGGCAC	OBlmT construction
PF/manA	CGCCATATGGACCGCCTCGCCAACAC	Amplification of *manA*
PR/manA	TGCACTGCAGGAACCGCCTTCAGACGGCGA	for OBlmT/ManAB construction
PF/manB	AAAACTGCAGGTGGCTGATCTGTCGCAGCT	Amplification of *manB* for OBlmT/ManAB
PR/manB	CTAGTCTAGATTCAAACCGTCGAGGG	construction

	C	
PF/blmT	CTAGTCTAGAGTGAGCTCCCTCGCCG	Amplification of *blmT*
	TCC	for OBlmT/ManAB
PR/blmT	ATTTGCGGCCGCTCCTCATCGTCGGG	construction
	CAC	
**Primers for RT-qPCR analysis**		
16s RNA-F	GGCCTATCAGCTTGTTGGT	Amplification of partial
16s RNA-R	CCGTCACTTTCGCTTCTTC	*16s RNA* sequence
dasR-F	CGGCTCAAACGTCATCTGT	Amplification of partial
dasR-R	CTGGGCCTTCATGTCCTCC	*dasR* sequence
dasA-F	ACCGAATCCCTCAACAAGC	Amplification of partial
dasA-R	TTGCCGTCCTTCTTCACCA	*dasA* sequence
dasB-F	TGGATCGGCTTCGACAACT	Amplification of partial
dasB-R	CGAACAGCCACTGGAAAAC	*dasB* sequence
dasC-F	CAACTCCTTCATCACCACG	Amplification of partial
dasC-R	TCCAGTTCCTTCGGCACAG	*dasC* sequence
dasD-F	GGACACGCTGACCCGAGACG	Amplification of partial
dasD-R	AGGTTGCCGGGGAAGGAGG	*dasD* sequence
nagB-F	TCTGCCAGTTGGACGAGTACG	Amplification of partial
nagB -R	TCGTTGAACCCGATGTGCC	*nagB* sequence
nagK-F	CGGGATAAAAGCCGCCCTCA	Amplification of partial
nagK-R	GGCTGTGCCCGTTGTCGTGT	*nagK* sequence
nagA-F	TCGCCACCCACCTCTTCAA	Amplification of partial
nagA-R	CGTACATGCCGTCGCTCAT	*nagA* sequence
blmR-F	CTTCCGGATGCTGGGGCA	Amplification of partial
blmR-R	TCCAGCAGCTCGTGCTGC	*blmR* sequence
blmT-F	CCCGCGACGTGATCTACTG	Amplification of partial
blmT-R	GGCACGTACTGCATGTAGC	*blmR* sequence
orf28-F	GTCCCTTACGAGGAGAAGCC	Amplification of partial
orf28-R	TGCACGTCGAAGAAGGTCAT	*orf28* sequence
blmX-F	TCACCTCAGCAGCGGCATT	Amplification of partial
blmX-R	CGTCCGGGTCATGGACGA	*blmX* sequence
blmV-F	CGCTTCCTGGACACCGACC	Amplification of partial
blmV-R	GCGCAGGCGCATGTAGCAGT	*blmV* sequence

blmC-F	TGAGCCGGGCCAAGGAACT	Amplification of partial
blmC-R	GGATGATGTCGGTGGTGGAG	*blmC* sequence
blmD-F	TGTTGCGGGAGGGCAGGAT	Amplification of partial
blmD-R	GCAGGCTCACCTCGTCGTT	*blmD* sequence
blmE-F	CATCCCATCCGCCTCCCA	Amplification of partial
blmE-R	TCGAGCGGCCATCTCCTGT	*blmE* sequence
blmF-F	CAGTGGGAGGTCGAGAAGTG	Amplification of partial
blmF-R	CGTCGTGAACGGCATGGTC	*blmF* sequence
blmG-F	AGCCACGGCATGGACATCA	Amplification of partial
blmG-R	AGCCGGATCAGCCCTTCGA	*blmG* sequence
manA-F	GCCGCTCACCGACGTCAT	Amplification of partial
manA -R	TGTGGTGCGGGTCCTTGTA	*manA* sequence
manB-F	CTATGTTCACGGCCAGCCA	Amplification of partial
manB -R	GTCCACGACGGTCTTCAGC	*manB* sequence
**Primers for β-galactosidase reporter system**		
pDasR/F	taagaaggagatataATGGCAACCGAGGGCG GGACA	Amplification of the whole *dasR* sequence
pDasR/R	gtggtggtggtggtgctcgagGCCTATTCCGACG CGGGCCG	

Table S3 List of intracellular metabolites detected by GC-MS

	Metabolites	KEGG NO.	Rank of VIP score
	**Glycometabolism**		
1	Glycerol	C00116	1.1593
2	D-Glycerate 3-phosphate	C00197	1.4209
3	D-Talose	C06467	0. 7434
4	Inositol	C00137	0. 6898
5	Lactose	C00243	0. 4391
6	Glucuronic acid	C00191	0.4906
7	Galactose	C00124	0. 4179
8	N-Acetyl-D-glucosamine	C00140	1.7747
	**Pyruvate metabolism and TCA**		
9	Lactate	C00256	1.0735
10	Acetaldehyde	C00084	1.071
11	Ethanol	C00469	0.3138
12	Fumarate	C00122	1.0719
13	Succinate	C00042	0.2657
14	Malic acid	C00149	1.4597
15	Oxaloacetate	C00036	0.4438
16	Pyruvic acid	C00022	1.4527
17	2-Oxoglutarate	C00026	1.1671
18	Fumarate	C00122	1.0719
	**Nucleotide metabolism**		
19	Oxalic acid	C00209	1.2202
20	Adenine	C00147	1.1951
21	Adenosine	C00212	0. 6942
22	Inosine	C00294	1.3095
23	Uracil	C00106	0. 3887
24	Uridine	C00299	1.6143
	**Amino acid metabolism**		
25	Serine	C00065	0.4339
26	Glycine	C00037	0. 7828
27	Sulfide	C00283	0. 4718

	Metabolites	KEGG NO.	Rank of VIP score
28	Histidine	C00135	1.9357
29	Cysteine	C00097	1.4269
30	Shikimate	C00493	1.02166
31	Tyramine	C00483	1.4083
32	Quinolinate	C03722	1.2535
33	Phenylalanine	C00079	1.2912
34	Benzoic acid	C00180	1.2394
35	Proline	C00148	0.9819
36	Cinnamic acid	C10438	0.8983
37	Indole	C00463	1.3752
38	Alanine	C00041	0. 4153
39	5-oxoproline	C01879	0. 6443
40	Urea	C00086	1.2008
41	Valine	C00183	0.4267
42	Leucine	C00123	1.5076
43	Aspartate	C00049	0. 6612
44	Methionine	C00073	1.1191
45	Lysine	C00047	0. 5629
46	Threonine	C00188	0.4583
47	Isoleucine	C00407	0. 6925
48	Glutamine	C00064	0. 3427
49	Ornithine	C00077	0.8167
50	Arginine	C00062	0.9887
	**Lipids metabolism**		
51	Linoleic acid	C01595	1.5301
52	1,3-propanediol	C02457	1.8752
53	Cholesterol	C00187	0. 2478
54	Lauric acid	C02679	0. 7437
55	Palmitic acid	C00249	1.1263
56	Stearic acid	C01530	1.3518
57	Arachidonic acid	C00219	1.0685
58	Hexanoic acid	C01585	0. 8377

	Metabolites	KEGG NO.	Rank of VIP score
59	Linolenic acid	C06427	0. 0485
	**Others**		
60	Benzamide	C09815	0.5837
61	Hydroxylamine	C00192	1.0285
62	Butanoic acid	C00246	0. 7091
63	Methanesulfonic acid	C11145	0. 9863
64	Boric acid	C12486	0. 7947
65	Phosphoric acid	C00009	0. 3632
66	2,4-dimethylaniline	C11003	0.5373
67	2-Hydroxyethylamine	C00189	0.9535
68	Monopalmitin		1.2785
69	Octadecane		0.2552
70	Glycerol monostearate		1.1912
71	Ginsenol		0. 7420
72	3-methoxyamphetamine		0.2947

Figure S1

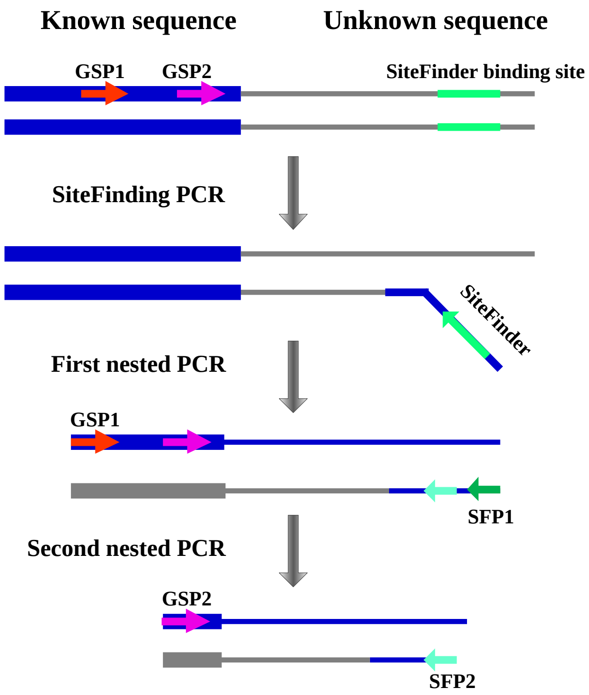

Figure S1 Detail schematic diagram of chromosome walking by SiteFinding PCR and nested PCR

Figure S2

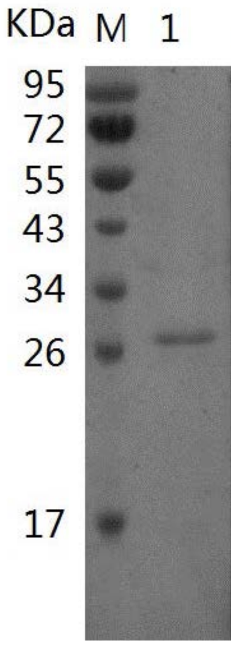

Figure S2 SDS-PAGE profile of 6×His-DasR used in EMSA assay. M: Protein marker; Lane 1: 6×His-DasR.

Figure S3

**P_{DasA}**	CCAAAAGGTCTAGGCCAAGCTGCGGGCACTGGTCTAAACCATTAAAGACCA<br>CTTTTCGGTCCTGAGGAGA
**P_{NagB}**	ACCTTCTCCCTGGGCCGGGCTCTGTTAGATTGGTCTATACCACCCCAATCCC<br>AAGATCGGCAGGCCCAGC
**P_{NagKA}**	CCGGACAGTTTCGCGCGTGGGGGTGGGAAAGGTCTAGTCCACTGTTCGACG<br>GGTGCGGGATGGGGGCGCC
**P_{BlmR}**	GAGAGCACTGTAAGCCCGAACCCGCAAGGATGACGAATTGCAAAATTGTGC<br>AAGTCGCTACATGATGGTCCGGCTGTGCCCGCAGGTAGCCGCGGGCACAGC<br>ACCAGACGCTGCCTCCGCGCACCGCGCGGGAGGCCCGGTGAGGCGAGAGG<br>CTGAGGTTCC
**P_{BlmR}-1**	TAAGCCCGAACCCGCAAGGATGACGAATTGCAAAATTGTGCAAGTCGCTAC<br>ATGATGGTCCGGCTGTGCC

Figure S3 DNA sequence of probes used in EMSAs. Probes P_{DasA}, P_{NagB} and P_{NagKA} respectively contained 70 bp promoter sequences of *dasA*, *nagB* and *nagKA*, the corresponding *dre* sites were marked in red font. P_{BlmR} included the whole promoter sequence of BlmR, and Probe PBlmR-1 contained 70 bp partial promoter sequence of BlmR in which the 12-2-12 bp binding site was underlined.

Figure S4

a

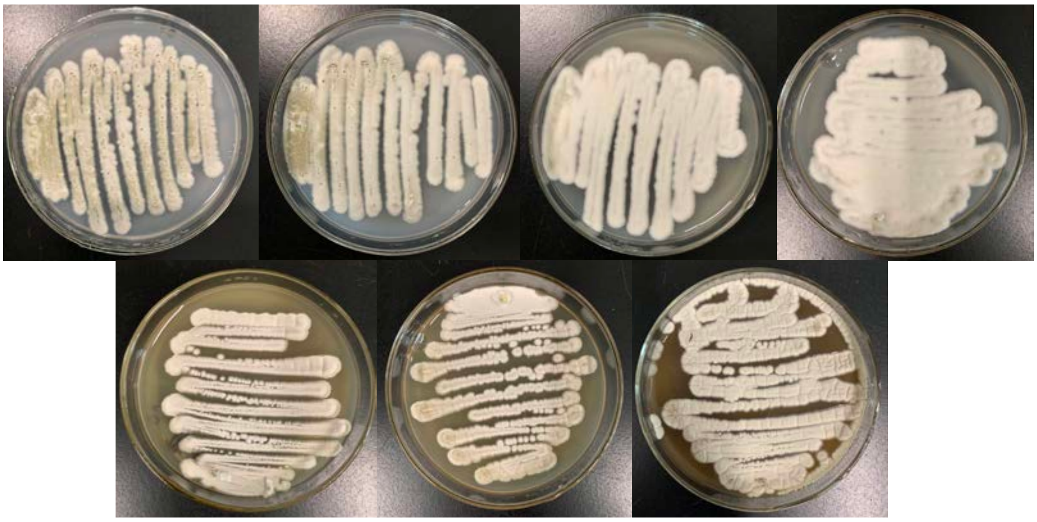

b

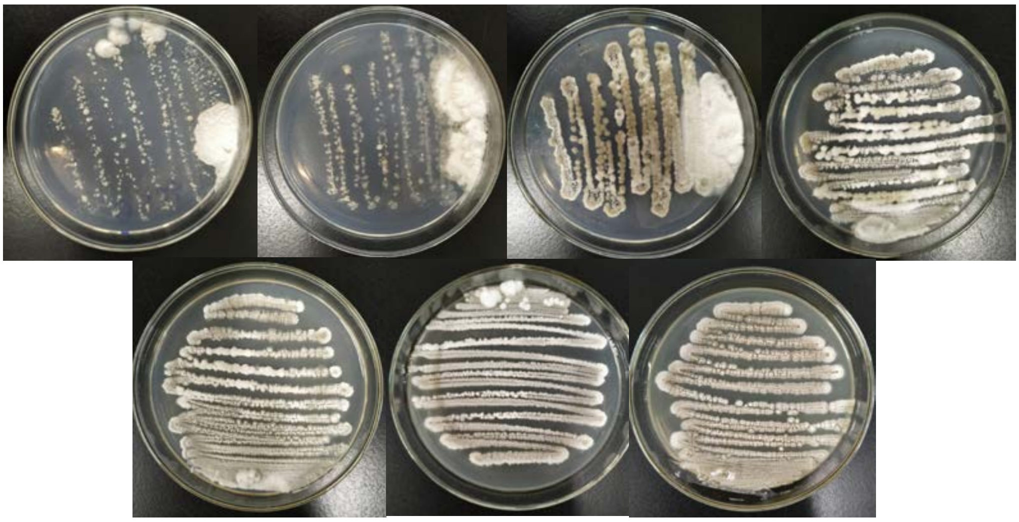

Figure S4 Effect of GlcNAc addition on spore morphogenesis in different mediums. **a** In the modified ISP4 solid medium, GlcNAc blocked the spores development and maturation. **b** In the MM solid plates, GlcNAc accelerated the spores under lower concentrations and played an negative effect on development under higher concentration (25g/L). GlcNAc concentration (left to right, top to bottom) in modified ISP4 and MM solid plates were 0, 2.5 g/L, 5 g/L, 10 g/L, 15 g/L, 20 g/L and 25 g/L.

Figure S5

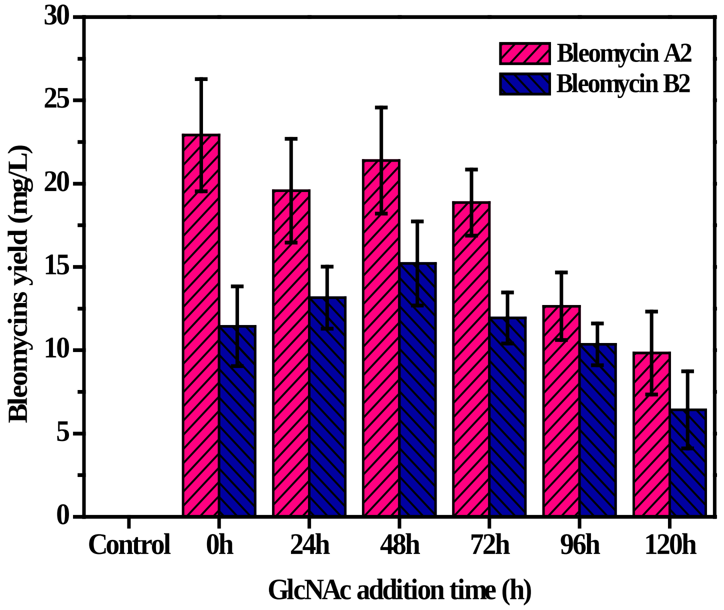

Figure S5 Fermentation characteristics of bleomycins in the different GlcNAc addition times. The control group was without GlcNAc.

References

1. Chen H, Wang J, Cui J, Wang C, Liang S, Liu H, Wen J: Negative regulation of bleomycins biosynthesis by ArsR/SmtB family repressor BlmR in *Streptomyces verticillus*. *Appl Microbiol Biotechnol* 2019, **103:**6629–6644.
